# The Pneumococcal Protein SufC Binds to Host Plasminogen and Promotes Its Conversion into Plasmin

**DOI:** 10.3390/microorganisms11122969

**Published:** 2023-12-12

**Authors:** Yoshihito Yasui, Satoru Hirayama, Takumi Hiyoshi, Toshihito Isono, Hisanori Domon, Tomoki Maekawa, Koichi Tabeta, Yutaka Terao

**Affiliations:** 1 Division of Microbiology and Infectious Diseases, Niigata University Graduate School of Medical and Dental Sciences, Niigata 951-8514, Japan; 2 Division of Periodontology, Niigata University Graduate School of Medical and Dental Sciences, Niigata 951-8514, Japan; 3 Center for Advanced Oral Science, Niigata University Graduate School of Medical and Dental Sciences, Niigata 951-8514, Japan

**Keywords:** *Streptococcus pneumoniae*, plasminogen, plasmin, autolysin, SufC

## Abstract

*Streptococcus pneumoniae* causes otitis media, sinusitis, and serious diseases such as pneumonia and bacteremia. However, the in vivo dynamics of *S. pneumoniae* infections and disease severity are not fully understood. In this study, we investigated pneumococcal proteins detected in the bronchoalveolar lavage fluid of an *S. pneumoniae*-infected mouse, which were assumed to be expressed during infection. Analysis of three proteins with unknown infection-related functions revealed that recombinant Fe-S cluster assembly ATP-binding protein (SufC) binds to the host plasminogen and promotes its conversion into plasmin. SufC was detected in the bacterial cell-surface protein fraction, but it had no extracellular secretory signal. This study suggests that *S. pneumoniae* releases SufC extracellularly through LytA-dependent autolysis, binding to the bacterial cell surface and host plasminogen and promoting its conversion into plasmin. The recruitment of plasmin by *S. pneumoniae* is considered useful for bacterial survival and spread, and SufC is suggested to facilitate this process.

## 1. Introduction

*Streptococcus pneumoniae* is a Gram-positive facultative anaerobic bacterium. Pneumococcal disease remains widespread, even with the availability of vaccines, and can be fatal, particularly in children and older adults. *S. pneumoniae* causes non-invasive illnesses, such as otitis media and sinusitis, and more serious conditions, such as bacterial pneumonia, bacteremia, and meningitis [1,2,3,4]. The prevalence of penicillin- and macrolide-resistant *S. pneumoniae* strains is increasing [5,6]. The mechanisms of pneumococcal infection in vivo need to be elucidated to prevent pneumococcal diseases and counter drug-resistant strain infections.

Pathogenic bacteria use host plasmin to promote infection and survival [7]. For example, pathogens utilize plasmin to degrade the extracellular matrix (ECM) covering host tissues and facilitate tissue invasion. This phenomenon has been observed mainly in groups A and B *Streptococcus,* and *S. pneumoniae* [8,9,10,11,12]. *S. pneumoniae* possesses proteins that function as receptors for anchoring plasminogens to bacterial cell surfaces [13]. Plasminogen recruited to the bacterial surface is converted into plasmin by the host tissue-type plasminogen activator (tPA) and/or urokinase-type plasminogen activator (uPA), allowing it to exhibit proteolytic activity [12,14].

We previously collected bronchoalveolar lavage fluid (BALF) from an *S. pneumoniae*-infected mouse and performed proteomic analysis using isobaric tags for relative and absolute quantitation (iTRAQ)–tandem mass spectrometry (MS/MS) [15] to detect pneumococcal proteins expressed in vivo. Although *S. pneumoniae* has 1911 protein-coding genes [16], only 15 pneumococcal proteins in BALF could be detected using iTRAQ-based proteome analysis (<1% of the total pneumococcal proteins) [15]. Six of the fifteen pneumococcal proteins reportedly possess plasminogen-binding activity [17], whereas the functions of the other nine molecules in infections are unknown.

In the present study, we hypothesized that a few of these nine proteins have a role in infection and analyzed their interactions with host proteins. We selected glutamate dehydrogenase (GdhA) [18], ribonuclease Y (Rny) [19], and the Fe-S cluster assembly ATP-binding protein (SufC) [20] for analysis because there are fewer reports on their effects on infection in other bacterial species.

## 2. Materials and Methods

### 2.1. Bacterial Strains and Culture Methods

*S. pneumoniae* strain D39 (wild-type) and its *lytA*-deleted (Δ*lytA*) [21] mutant strain were inoculated into Todd–Hewitt broth (Becton, Dickinson, Franklin Lakes, NJ, USA) supplemented with 0.5% yeast extract (THY) and precultured at 37 °C until the optical density at 600 nm (OD_600_) reached 0.1. The culture was subsequently added to fresh THY medium at a ratio of 1:200 and incubated statically at 37 °C. To prepare recombinant GdhA (rGdhA) and Rny (rRny), *Escherichia coli* DH5α and BL21 were used. *E. coli* strains were cultured in LB broth (10.0 g/L tryptone, 5.0 g/L yeast extract, 10.0 g/L NaCl, adjusted to pH 7.0, supplemented with 100 µg/mL ampicillin) or LB agar (LB broth with 15 g/L agar and 100 µg/mL ampicillin). To express recombinant SufC (rSufC), *Brevibacillus choshinensis* HPD31-SP3 was cultivated in 2SYNm broth (20.0 g/L glucose, 40.0 g/L Bacto soytone, 5.0 g/L Bacto yeast extract, 0.15 g/L CaCl_2_·2H_2_O, adjusted to pH 7.2, with 50 µg/mL neomycin), TMNm broth (10.0 g/L glucose, 10.0 g/L Phytone peptone, 5.75 g/L 35% Ehrlich bonito extract, 2.0 g/L yeast extract, 10 mg/L FeSO_4_·7H_2_O, 10 mg/L MnSO_4_·4H_2_O, 1 mg/L ZnSO_4_·7H_2_O, adjusted to pH 7.0, with 50 µg/mL neomycin), or MTNm agar (20 mM MgCl_2_ and 15 g/L agar added to TMNm broth).

### 2.2. Construction of rGdhA and rRny

rGdhA and rRny were prepared according to a previously described method [17]. DNA fragments encoding GdhA and Rny (*SPD_1158* and *SPD_1549*, respectively) were amplified from *S. pneumoniae* strain D39 genomic DNA via PCR using the primers listed in Table 1. PCR was conducted using KOD -Plus- Ver. 2 DNA polymerase (Toyobo, Osaka, Japan) according to the manufacturer’s guidelines. After confirming correct amplification via agarose gel electrophoresis, the PCR products were purified using a QIAquick PCR Purification Kit (QIAGEN, Hilden, Germany). The plasmid pQE-30-lacI^q^ [17] and the amplified DNA fragments were treated with the restriction enzymes BamHI-HF and KpnI-HF (New England Biolabs, Ipswich, MA, USA). The pQE-30-lacI^q^ and the respective insert DNA were mixed at a 1:5 molar ratio and ligated using Ligation High Ver.2 (Toyobo) following the manufacturer’s instructions. The ligated plasmid was introduced into *E. coli* DH5α competent cells (Takara Bio, Shiga, Japan). Ampicillin-resistant transformants were selected on ampicillin-supplemented LB agar. A few colonies were inoculated into ampicillin-supplemented LB medium and incubated at 37 °C with shaking. Plasmids were extracted from cultured bacteria using the QIAprep Spin Miniprep Kit (QIAGEN). Plasmid DNA was sequenced by Eurofins Genomics (Tokyo, Japan). The primers used for sequencing are listed in Table 1. Plasmids with error-free sequences were selected and introduced into *E. coli* BL21 cells.

*E. coli* BL21 cells expressing rGdhA or rRny were cultured in LB broth supplemented with ampicillin at 37 °C with shaking until the OD_600_ reached 0.6. Isopropyl β-d-thiogalactopyranoside (IPTG) was added (final concentration: 1 mM). The culture was again incubated at 37 °C with shaking for 5 h. The bacteria were pelleted via centrifugation (8000× *g* for 20 min at 4 °C), resuspended in binding buffer (20 mM NaHPO_4_, pH 7.4, 0.5 M NaCl, and 40 mM imidazole), and treated with 1 mg/mL lysozyme and 30 U/mL benzonase. The bacteria were lysed in an ice-water bath with six cycles of 10 s sonication followed by a 10 s pause. After the bacteria were sonicated, the supernatant was collected via centrifugation (10,000× *g* for 30 min at 4 °C) and filtered through a 0.22 µm polyvinylidene fluoride (PVDF) filter. Sepharose 6 Fast Flow (Cytiva, Marlborough, MA, USA) was adjusted to a 50% slurry concentration via resuspension in binding buffer. Thereafter, 1.5 mL of the slurry was added to a chromatography column (Bio-Rad, Hercules, CA, USA), and the flow-through was discarded. The sonicated supernatant was then added to the column and incubated for 1 h at 25 °C with rotation. After the incubation, the flow-through was discarded. The column containing the protein of interest was washed four times with binding buffer and eluted four times with 500 µL of elution buffer (20 mM NaHPO_4_, pH 7.4, 0.5 M NaCl, and 500 mM imidazole). The extracts were desalted and replaced with phosphate-buffered saline (PBS) using a PD-10 column (GE Healthcare, Chicago, IL, USA).

### 2.3. Construction of rSufC

rSufC was produced using the *Brevibacillus* Expression System (Takara Bio). The *sufC* gene (*SPD_0762*), without an initiation codon, was amplified from *S. pneumoniae* D39 genomic DNA using primers containing a sequence identical to that of the pBIC1 vector (Table 1). PCR, agarose gel electrophoresis, and PCR fragment purification were performed as described above. The pBIC1 vector was mixed with the purified PCR product at a 1:2 molar ratio and introduced into *Brevibacillus* competent cells. Neomycin-resistant transformants were selected on MTNm agar and inoculated into 2SYNm medium at 37 °C with shaking at 120 rpm. The bacteria were harvested using centrifugation (8000× *g* for 15 min at 4 °C), after which, the plasmids were extracted and sequenced. *B. choshinensis* HPD31-SP3 expressing rSufC was continuously agitated in TMNm medium at 32 °C with shaking at 120 rpm for 64 h. The supernatant was collected using centrifugation (10,000× *g* for 10 min at 4 °C), filtered through a 0.22 µm PVDF filter, and treated with 0.5 M NaCl and 40 mM imidazole. Sepharose 6 Fast Flow and chromatography columns were adjusted as described above, and the supernatant was added. rSufC was eluted and replaced with PBS as described above.

### 2.4. SDS-PAGE and Western Blotting

Sodium dodecyl sulfate–polyacrylamide gel electrophoresis (SDS-PAGE) and Western blotting were performed as previously described with slight modifications [15,22]. Acrylamide gels were prepared using TGX FastCast Acrylamide Kit 12% (Bio-Rad), and the samples were subjected to SDS-PAGE. After electrophoresis, the gels were stained with Coomassie Brilliant Blue (CBB) using Quick-CBB (Fujifilm Wako Pure Chemical, Osaka, Japan). For Western blotting, proteins from the post-electrophoresis gels were electroblotted on PVDF membranes. The membranes were blocked with 1% skim milk in Tris-buffered saline containing 0.05% Tween-20 (TBST). The membranes were rinsed with TBST and subsequently treated with anti-SufC antiserum (prepared by immunizing rabbits with a peptide (NH_2_-C+AMNAGKEDDEKISV-COOH), subcontracted to Eurofins Genomics). Secondary modifications were performed using horseradish peroxidase (HRP)-conjugated goat anti-rabbit IgG (Cell Signaling Technology, Beverly, MA, USA). The substrate (ECL Select Western Blotting Detection Reagent, Cytiva) was added, and chemiluminescence was detected using an ImageQuant LAS-4000 Mini (Fujifilm, Tokyo, Japan).

### 2.5. ELISA

The binding of rGdhA, rRny, and rSufC to host proteins or bovine serum albumin (BSA) was analyzed using an enzyme-linked immunosorbent assay (ELISA) with minor modifications of previous methods [15,22]. Plasminogen, laminin, fibronectin, and fibrinogen from human plasma (Sigma-Aldrich, St. Louis, MO, USA) and elastin from human lungs (Elastin Products Company, Owensville, MO, USA) were used as host proteins. The host proteins and BSA were diluted with a coating buffer (1.59 g/L Na_2_CO_3_, 2.93 g/L NaHCO_3_, 0.2 g/L NaN_3_, adjusted to pH 9.6). The samples were added to 96-well Half Area Clear Flat Bottom Polystyrene High Bind Microplates (Corning, Corning, NY, USA) at 1 µg/well and incubated overnight at 4 °C. The coated wells were filled with 1% skim milk in PBS containing 0.05% Tween-20 (PBST) and incubated at 37 °C for 2 h for blocking. After washing three times with PBST, 1 µg each of rGdhA, rRny, and rSufC was added and incubated for 1 h at 37 °C. After the wells were washed three times with PBST, anti-GdhA (1:1000), anti-Rny (1:5000) (prepared by immunizing rabbits with peptides (NH_2_-C+VKEKRRARLTEYAA-COOH or NH_2_-C+IREAEQEVKERSDK-COOH, respectively), subcontracted to Eurofins Genomics), or anti-SufC (1:10,000) antiserum diluted in PBST containing 0.5% skim milk was added to the wells and incubated at 37 °C for 1 h. The wells were washed with PBST and incubated with alkaline phosphatase-linked anti-rabbit IgG (H + L) (Bethyl Laboratories, Montgomery, TX, USA) diluted 1:5000 in PBST containing 0.5% skim milk at 37 °C for 1 h. After washing with PBST, 3 g/L disodium *p*-nitrophenylphosphate hexahydrate in diethanolamine buffer (9.7 mL/L diethanolamine, 0.2 g/L NaN_3_, 0.1 g/L MgCl_2_·6H_2_O in water, adjusted to pH 9.6) was added and incubated at 37 °C for 30 min. Absorbance at 405 nm (*A*_405_) was measured using a microplate spectrophotometer (Multiskan FC, Thermo Fisher Scientific, Waltham, MA, USA). For the binding analysis of rSufC to plasminogen, ε-aminocaproic acid (EACA, Sigma-Aldrich) diluted in 0.5% skim milk was added, and ELISA was performed.

### 2.6. Binding Analysis Assay Using Surface Plasmon Resonance

The analysis of rSufC binding to human plasminogen or BSA using surface plasmon resonance (SPR) was adapted from previous methods [15,23,24]. A Biacore X100 instrument (GE Healthcare) was used to measure binding. rSufC was first diluted in 20 mM sodium acetate buffer (pH 3.7) to 50 µg/mL and then immobilized on a Series S Sensor Chip CM5 (Cytiva). Human plasminogen and BSA were diluted in running buffer (100 mM HEPES, pH 7.4, containing 150 mM NaCl, 3 mM EDTA, and 0.005% surfactant P20) and allowed to flow into the flow cell at 25 °C. The flow rate was set at 10 µL/min for immobilization and 20 µL/min for binding analysis. The sensor chips were replaced with 50 mM HCl. Experimental data were processed using the Biacore X100 evaluation software (GE Healthcare).

### 2.7. Plasminogen Activation Analysis

Plasminogen activation was analyzed as previously described [15]. Briefly, 5–40 pmol of rSufC and 2 µg of plasminogen were preincubated in 96-well microtiter plates at 37 °C for 30 min. Thereafter, 0.1 µg of recombinant human tPA (NKMAX, Sungnam, Republic of Korea) and 0.45 µmol of S-2251 substrate (DiaPharma Group, West Chester, OH, USA) were added to the wells and incubated at 37 °C. Coloration upon the degradation of S-2251 due to plasmin was assessed every 10 min by measuring the *A*_405_ using a microplate spectrophotometer (Multiskan FC, Thermo Fisher Scientific).

### 2.8. rSufC Interaction with Plasmin

The interaction between rSufC and plasmin was analyzed as previously described [15] with certain modifications. Briefly, 3 µg of human-plasma-derived plasmin (Fujifilm Wako Pure Chemical) and rSufC were mixed in 50 µL of PBS and incubated at 37 °C for 3 h. The samples were separated via SDS-PAGE, as described above, and stained with CBB.

### 2.9. Analysis of Bacterial Surface Proteins

Bacterial surface proteins were extracted as previously described [15,25,26,27]. The wild -type and Δ*lytA* of *S. pneumoniae* strain D39 were cultured in THY medium for 12 h, collected via centrifugation (8000× *g* for 10 min at 4 °C), washed with PBS, and then suspended in 8 M urea. The suspension was incubated at 25 °C for 1 h with stirring, and the supernatant obtained after centrifugation (15,300× *g*, for 5 min at 4 °C) was used as the surface protein fraction. The samples were analyzed using Western blotting according to the procedure described above.

### 2.10. Real-Time PCR

The wild-type and Δ*lytA* of *S. pneumoniae* strain D39 were cultured in THY medium for 4–12 h. Approximately 10^9^ bacterial cells were collected via centrifugation (8000× *g* for 10 min at 4 °C), suspended in NucleoSpin RNA Buffer RA1 (MACHEREY-NAGEL, Allentown, PA, USA), transferred to Lysing Matrix B (MP Biomedicals, Irvine, CA, USA), and homogenized with a MagNA Lyser (Roche Diagnostics K.K., Basel, Switzerland). Total RNA was extracted according to the NucleoSpin RNA instructions. The extracted RNA was reverse-transcribed using ReverTra Ace qPCR RT Master Mix with gDNA Remover (Toyobo). Real-time PCR was performed with cDNA samples and Premix Ex Taq (Probe qPCR) (Takara) using a StepOnePlus Real-Time PCR System (Applied Biosystems, Waltham, MA, USA) according to the manufacturer’s instructions. The primers and probes used are listed in Table 1. The *sufC* mRNA transcript levels were normalized to the 16S rRNA transcript levels.

### 2.11. Statistical Analysis

Statistical analyses were performed using Prism 9 version 9.5.1 (GraphPad Software, La Jolla, CA, USA). The data were statistically evaluated using one-way analysis of variance and Tukey’s multiple comparison test for ELISA and real-time PCR and two-way analysis of variance and Sidak’s multiple comparison test for plasminogen activation analysis. Differences were considered statistically significant at *p* < 0.05.

## 3. Results

### 3.1. rSufC Binds to Plasminogen, Which Is Inhibited by Lysine Analogs

**Figure 1:**
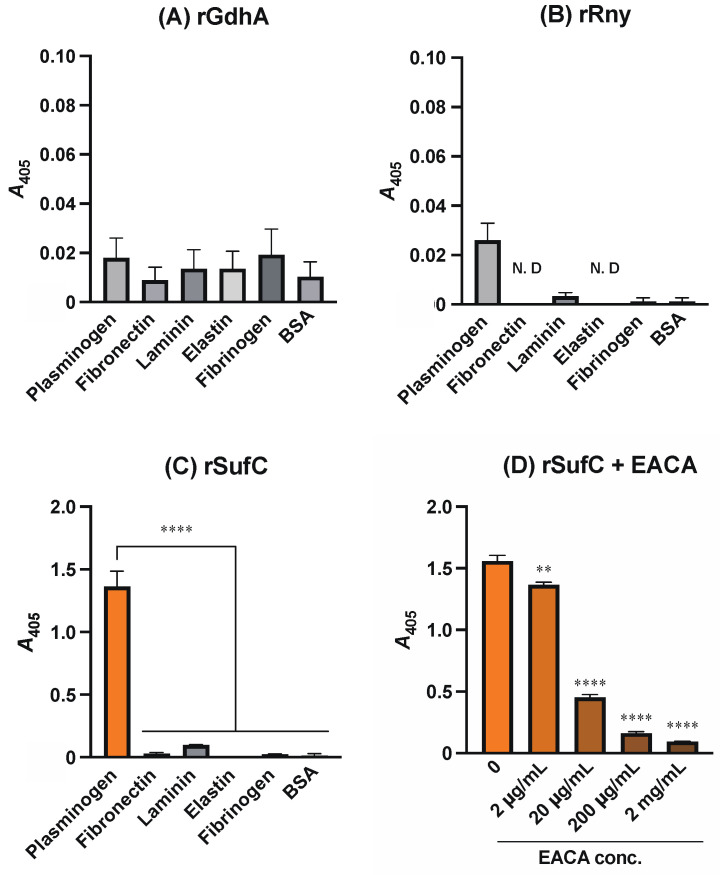
Binding of pneumococcal recombinant proteins to host proteins. The binding of (**A**) rGdhA, (**B**) rRny, and (**C**) rSufC to each host protein (plasminogen, fibronectin, laminin, elastin, and fibrinogen) and BSA was measured using ELISA. After incubating each recombinant protein in the wells of each host-protein-coated microtiter plate, the bound recombinant protein was detected using antiserum against each pneumococcal protein and secondary antibody. Asterisks indicate significant differences between groups (**** *p* < 0.0001). N.D. indicates not detectable. (**D**) Inhibition of rSufC binding to plasminogen by EACA. ELISA results were obtained by adding EACA with rSufC to the wells of a plasminogen-coated microtiter plate. Asterisks indicate significant differences compared with the zero group (** *p* < 0.01, **** *p* < 0.0001). Error bars indicate the standard error (*n* = 3).

We produced rGdhA, rRny, and rSufC using *E. coli* or *B. choshinensis*. The binding of rGdhA, rRny, and rSufC to various host proteins (plasminogen, laminin, fibronectin, elastin, and fibrinogen) and BSA was measured using ELISA. While rSufC was significantly bound only to plasminogen, rGdhA and rRny did not bind to either host protein (Figure 1A–C). The binding of plasminogen to other proteins may involve a lysine binding site. Therefore, when ε-aminocaproic acid (EACA), a lysine analog, was added, and ELISA was performed, the binding of rSufC to plasminogen was inhibited dose-dependently (Figure 1D).

### 3.2. Detailed Analysis of the Binding of rSufC to Plasminogen Using SPR

**Figure 2:**
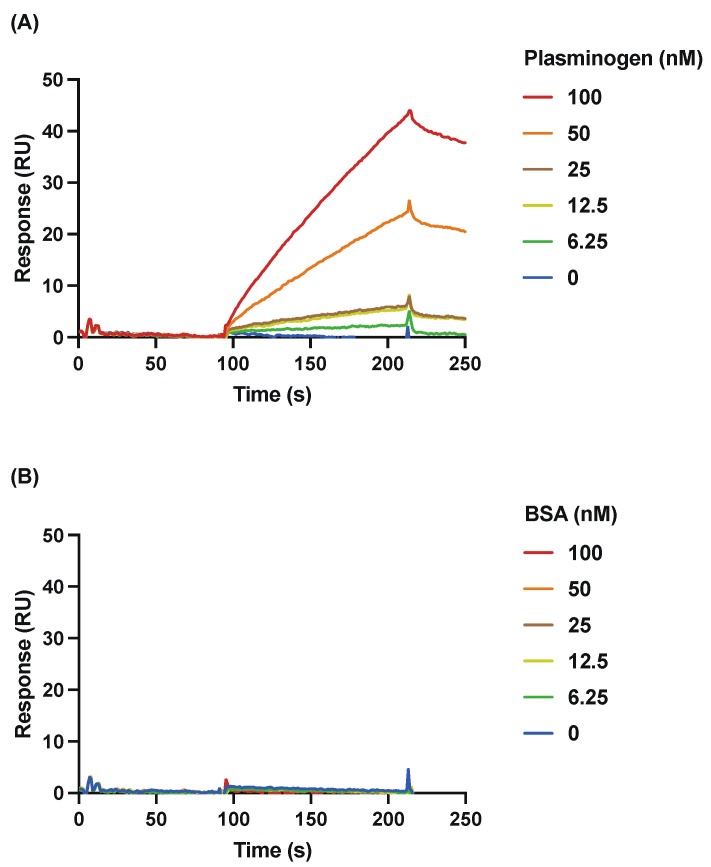
Binding activity of rSufC to plasminogen and BSA. The binding of rSufC to (**A**) human plasminogen and (**B**) BSA was analyzed using SPR. Plasminogen and BSA were prepared and applied at concentrations ranging from 6.25 to 100 nM and reacted with rSufC immobilized on the sensor chip at the 1000 RU immobilization level.

The interaction of rSufC with plasminogen was analyzed in detail using SPR. Plasminogen bound to the biosensor-bound rSufC in a dose-dependent manner (Figure 2A). The apparent association rate (*k_a_*), dissociation rate (*k_d_*), and dissociation constant (K_D_) of rSufC with plasminogen were 1.958 × 10^4^ 1/Ms, 1.558 × 10^−3^ 1/s, and 7.953 × 10^−8^ M, respectively. In contrast, BSA did not bind to rSufC at the same dose as plasminogen; consequently, the *k_a_*, *k_d_*, and K_D_ of BSA could not be calculated (Figure 2B).

### 3.3. rSufC Promotes Plasminogen Activation

**Figure 3:**
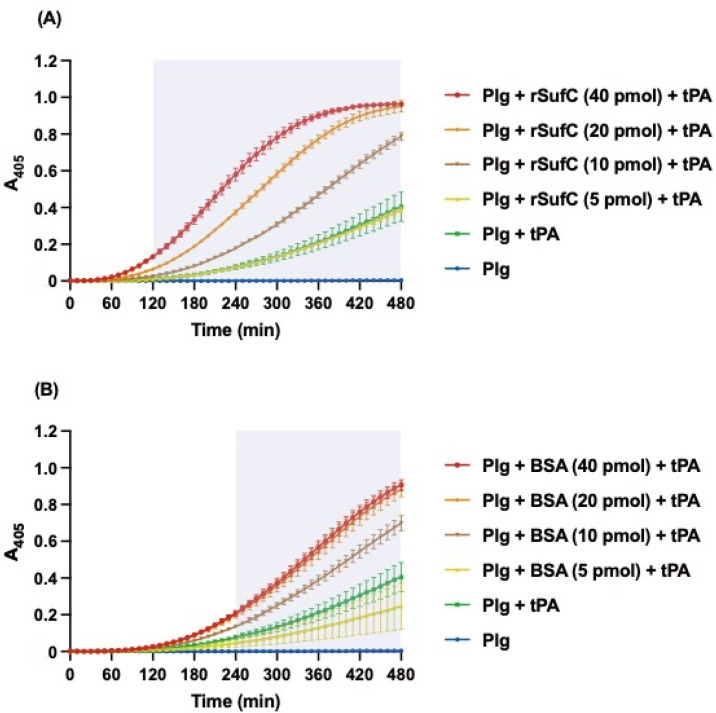
rSufC promotes plasminogen activation due to tPA. Plasminogen (2 µg) was preincubated with 5–40 pmol of (**A**) rSufC or (**B**) BSA and then incubated with 100 ng of tPA and the chromogenic substrate S-2251 at 37 °C. The *A*_405_ was measured every 10 min. The filled background indicates areas that were significantly higher when preincubated with 40 pmol of rSufC or BSA than when preincubated with Plg (plasminogen) + tPA. Error bars indicate the standard error (*n* = 3).

Plasminogen is activated by tPA. We investigated whether the binding of rSufC to plasminogen affects plasminogen activation. Plasmin converted from plasminogen was quantitatively analyzed using a chromogenic substrate. Plasminogen was activated by the addition of tPA, and the absorbance of the solution increased in a time-dependent manner as the plasmin substrate was degraded. The preincubation of plasminogens with rSufC or BSA significantly enhanced plasminogen activation due to tPA in a dose-dependent manner (Figure 3A,B). The promotion of plasminogen activation by tPA was significantly higher with rSufC than with BSA. These results indicate that rSufC binds to plasminogen and promotes plasminogen activation in plasmin.

### 3.4. rSufC Is Degraded by Plasmin

**Figure 4:**
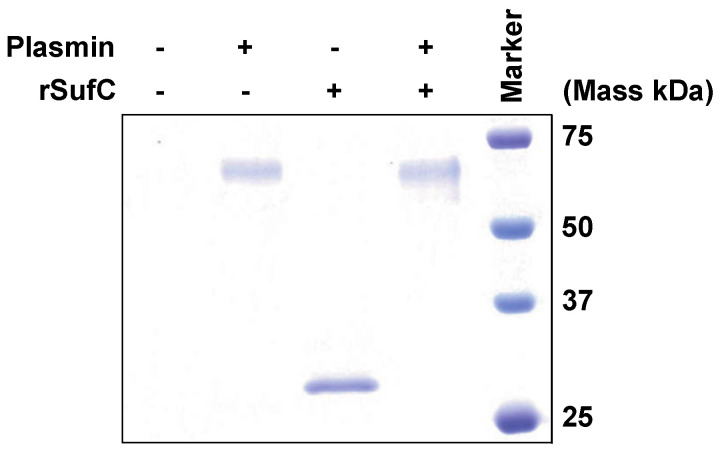
Degradation of rSufC due to plasmin. Plasmin (3 µg), rSufC (3 µg), and their mixtures (3 µg each) were incubated in PBS at 37 °C for 3 h. They were subjected to SDS-PAGE and stained with CBB. Precision Plus Protein Dual Color Standards (Bio-Rad) were used as markers.

The interaction between rSufC and plasmin was analyzed. rSufC and plasmin were incubated, and each protein in the mixture was subjected to SDS–PAGE. When plasmin or rSufC alone was incubated, each protein was detected at the correct molecular weight (Figure 4). No rSufC bands were detected when the plasmin and rSufC were mixed and incubated. Therefore, rSufC was degraded by the proteolytic activity of plasmin.

### 3.5. LytA-Mediated Autolysis Has a Profound Effect on the Extracellular Release of SufC in S. pneumoniae

**Figure 5:**
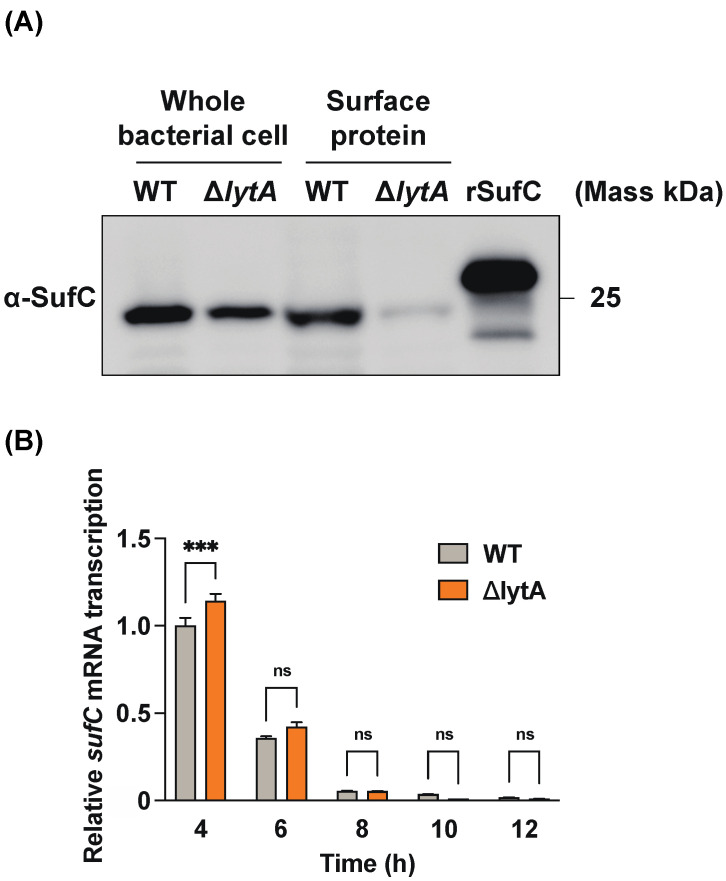
SufC detection and *sufC* mRNA transcript level analysis in the wild type and Δ*lytA* mutant of *S. pneumoniae* strain D39. (**A**) SufC detection in the wild-type (WT) and its Δ*lytA* mutant strains cultured in THY medium for 12 h. Whole bacterial cell samples and surface protein fractions were collected. Whole-cell bacterial samples, surface protein fractions, and rSufC (200 ng) were subjected to SDS-PAGE, and the proteins were electroblotted onto PVDF membranes. The membranes were incubated with an anti-SufC peptide antiserum (α-SufC, 1:3000 dilution) and then with an HRP-conjugated secondary antibody (1:3000 dilution). Chemiluminescence due to the enzymatic activity of HRP was detected after an exposure time of 8 s. (**B**) Analysis of *sufC* mRNA transcription over time in WT and Δ*lytA* strains. Both mRNA transcript levels were normalized to the 16S rRNA transcript level and expressed relative to the transcript level after 4 h of culture in the WT strain. Error bars indicate the standard error (*n* = 3). Asterisks indicate significant differences (*** *p* < 0.001). No significant difference is indicated by ns.

We investigated the mechanism underlying the release of SufC from pneumococcal cells. As SufC is a Fe-S cluster ATPase that functions only inside bacterial cells, it has no signal sequence for extracellular secretion. *S. pneumoniae* is a unique bacterium that autolyzes by LytA, indicating that intracellular proteins may be released extracellularly via LytA-induced autolysis. The surface layer proteins of the wild-type and Δ*lytA* mutant strains were collected, and SufC was detected using Western blotting. Peptide antiserum detected SufC bands from wild-type and Δ*lytA* mutant cells (Figure 5A). However, the SufC band was detected only in the surface proteins of the wild type, suggesting that LytA-induced bacterial cell lysis releases SufC, which localizes to the bacterial surface. Real-time PCR results indicated that the *sufC* mRNA levels were higher in the Δ*lytA* strain than in the wild-type strain at 4 h and the same at other time points (Figure 5B). Nevertheless, SufC was detected on the surface of the wild-type strain. Thus, SufC was released into the culture supernatant by LytA-induced bacterial cell lysis and bound to the bacterial surface layer.

## 4. Discussion

**Figure 6:**
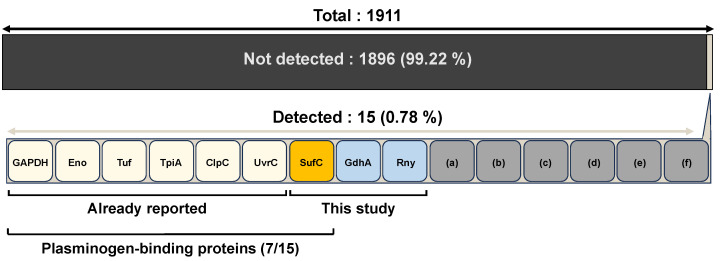
Types and numbers of proteins identified in BALF from a mouse model of pneumococcal pneumonia. Proteome analysis was performed using iTRAQ-MS/MS. The Kyoto Encyclopedia of Genes and Genomes (KEGG) database lists 1911 protein-coding genes for *S. pneumoniae* strain D39 [16]. This proteome analysis identified 15 proteins from BALF. Six of these proteins have been shown to have plasminogen-binding capacity. In this study, we analyzed three proteins, GdhA, Rny, and SufC, and found that SufC is a plasminogen-binding protein. Unanalyzed pneumococcal proteins are shown in (a)–(f): (a) putative transcriptional regulator, (b) ribosomal protein L7/L12, (c) ribosomal protein L29, (d) fructose-1,6-bisphosphate aldolase, (e) ATP synthase F1 beta subunit, and (f) ABC transporter, transmembrane protein Vexp3.

In a previous study, we performed an iTRAQ-MS/MS proteomic analysis of BALF from a mouse model of pneumococcal pneumonia and detected 15 different pneumococcal proteins (Figure 6) [15]. The proteins detected in the in vivo samples represented less than 1% of the proteins present in *S. pneumoniae* strain D39, suggesting that they were expressed during infection. Three of fifteen pneumococcal molecules (glyceraldehyde-3-phosphate dehydrogenase (GAPDH), α-enolase (Eno), and elongation factor Tu (Tuf)) have been reported to possess plasminogen-binding activity [28,29,30,31,32,33,34,35,36]. Furthermore, we recently showed that three other proteins (triosephosphate isomerase (TpiA), ATP-dependent Clp protease ATP-binding subunit ClpC, and excinuclease ABC subunit C (UvrC)) have a high affinity for plasminogen [15,17]. Among the proteins detected in the BALF of an *S. pneumoniae*-infected mouse, we focused on three proteins (GdhA, Rny, and SufC) that have not yet been reported to have a role in infection. We prepared these recombinant proteins and analyzed their affinity for host proteins.

Binding analysis of each recombinant protein to various host proteins using ELISA and SPR revealed that rSufC binds only to human plasminogen (Figure 1 and Figure 2). Neither rGdhA nor rRny exhibited affinity for the host proteins tested (Figure 1). The binding of rSufC to plasminogen was inhibited by the lysine analog EACA in a dose-dependent manner, suggesting that the lysine residues constituting SufC were involved in their binding (Figure 1D). Plasminogen is converted into plasmin by tPA. In this reaction, pre-incubation of plasminogen with rSufC promoted conversion in a dose-dependent manner (Figure 3). BSA promotes conversion into plasmin in a dose-dependent manner. However, rSufC required less time to achieve a significant conversion enhancement. One possibility is that BSA stabilizes the enzymatic reaction because it is sometimes added to the reaction system of restriction enzymes, thereby contributing to its ability to promote plasmin conversion. BSA did not bind plasminogen or rSufC (Figure 1 and Figure 2). In contrast, rSufC was degraded by plasmin (Figure 4). SufC is an ATPase that functions within bacterial cells but lacks a signal sequence for extracellular secretion [37]. However, SufC was released from the bacterial cells via pneumococcal autolysis and adhered to the bacterial cell surface (Figure 5A). Because pneumococcal autolysis is regulated by LytA [38], analysis of the bacterial surface protein fractions of the Δ*lytA* strain showed that the SufC amount was less than that of the wild-type strain (Figure 5A). Here, the *sufC* mRNA transcript level in the Δ*lytA* strain was either higher than or did not differ from that in the wild-type strain, confirming that *lytA* gene deletion did not affect the mRNA transcript level (Figure 5B). Therefore, SufC is released extracellularly via the LytA-dependent autolysis of *S. pneumoniae* and exhibits plasminogen-binding activity distinct from its function as an enzyme in bacterial cells.

Fe-S clusters play several important roles in biological activities, including respiration, photosynthesis, and DNA repair [39,40]. The Fe-S cluster was configured using the Suf system, which is encoded by the *sufABCDSE* operon in bacteria [37]. SufC is a component of the Suf system with ATPase activity and forms a complex with SufB and SufD during Fe-S cluster biosynthesis [41]. The *suf* operon is widely distributed in prokaryotes, including archaea, and has been observed in plant chloroplasts [42,43]. SufC is involved in the growth of *Salmonella enterica* serovar Typhi [44]. However, there are no reports regarding its role in infection.

**Figure 7:**
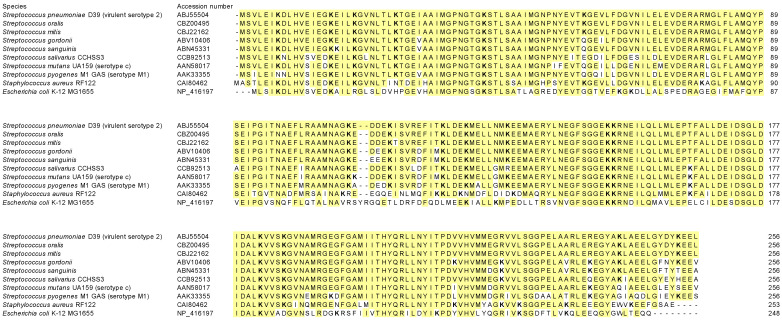
SufC amino acid sequence alignment for each species. The alphanumeric characters in the second column indicate accession numbers. The highlighted sequences were consistent with those of *S. pneumoniae* D39. Lysine residues are indicated in bold.

Among the proteins expressed by *S. pneumoniae*, Eno [28,29,30,31,32,33,34], GAPDH [35], choline-binding protein E (CbpE) [45], plasmin- and fibronectin-binding protein A (PfbA) [46], plasminogen- and fibronectin-binding protein B (PfbB) [47], endopeptidase O (PepO) [48], Tuf [36], phosphoglycerate kinase [49], pneumococcal surface protein C (PspC) [50], TpiA [15,51], ClpC [17], and UvrC [17] bind plasminogen. A few of these, including Eno [28], CbpE [45], PepO [48], Tuf [36], and TpiA [15], use lysine residues in the protein molecule to bind to plasminogen. A recent study on TpiA reported that the plasminogen binding site was located at the C-terminus [51]. In Eno, which has been studied the most, the binding site for plasminogen has been identified as two C-terminal lysine residues and the nine-amino acid motif FYDKERKVY, located at positions 248–256 [30]. Although CbpE has no lysine residues at the end of its amino acid sequence, the surface-exposed lysine residues 259, 267, and 319 are responsible for binding to plasminogen [45]. SufC, the target of this study, does not contain lysine residues at its C-terminus or a plasminogen-binding motif as observed in Eno. However, a lysine residue may be involved in plasminogen binding. Thus, similar to CbpE, a sterically accessible lysine residue may be involved in plasminogen binding. The high homology in the amino acid sequence of SufC between *S. pneumoniae* and several other bacterial species suggests that it may facilitate plasmin activation in bacteria other than *S. pneumoniae* (Figure 7 and Table 2).

Plasminogen is secreted into the blood as a glycoprotein and is converted into plasmin by tPA and/or uPA. Plasmin plays an important role in the fibrinolytic system by degrading fibrin and dissolving thrombi [52]. Because plasmin exhibits high proteolytic activity, it is involved in several other functions, such as tissue remodeling and inflammation, as well as thrombolysis [53]. Although plasminogen and plasmin have physiological functions in the host, bacteria can utilize them to facilitate infections [13]. For example, plasmin degrades host ECM components, such as laminin and fibronectin, and helps spread bacteria into host tissues [54]. *S. pneumoniae* promotes the adhesion of epithelial and endothelial cells by binding to plasminogen [55]. Furthermore, plasmin activation on the bacterial cell surface degrades intercellular junctions, enabling the invasion of the pericellular tissue [55]. In addition, plasmin prevents phagocytosis due to neutrophils via bacterial opsonization by degrading complement C3b and IgG [56]. Plasmins also favor bacterial survival by degrading extracellular histones, which have potent antimicrobial activity [57].

## 5. Conclusions

SufC, an *S. pneumoniae* protein involved in Fe-S cluster biosynthesis in bacterial cells, was released from bacteria via autolysis and bound to host plasminogen for the first time. Many pneumococcal proteins detected in the BALF of an *S. pneumoniae*-infected mouse model have plasminogen-binding abilities, and the expression of multiple such proteins during infection is intriguing, suggesting the importance of plasminogen-binding ability in infection.

## Figures and Tables

**Table 1:** The primers and probes used in this study.

Name	Sequence (5′-3′)	Reference
gdhA_BamHI_F	CTACGGATCCACATCTGCTAAAGAATATATCCAAAGC	This study
gdhA_KpnI_R	CTCGGGTACCTTAAACAATACCTTGTGCAATCATAG	This study
rny_BamHI_F	TCTAGGATCCGAAATCATGTCGCTTGCG	This study
rny_KpnI_R	TCTAGGTACCTTATTTAGCATAATCTACTGCACGAAG	This study
pQE30-ins-F	CGCGATATCAGGATTCGG	[17]
pQE30-ins-R	CTGAACAAATCCAGATGGAG	[17]
pBIC_sufC_F w/o ATG	TCAGTATTAGAGATCAAAGATCTTCACG	This study
pBIC_sufC_R	GATACGAGGGAATTACAATTCTTCC	This study
pBIC-ins-F	CGCGATATCAGGATTCGG	[15]
pBIC-ins-R	CAATGTAATTGTTCCCTACCTGC	[15]
sufC_qPCR_F	TCATCACTCACTACCAACGTCTTTT	This study
sufC_qPCR_R	GTTCTTCAGCTAATTTTGCGTATCCT	This study
sufC_qPCR_probe	(FAM) GGTCGTGTTGTCCTTTCTGGT (TAMRA)	This study
16S_qPCR_F	TCCTACGGGAGGCAGCAGT	[17]
16S_qPCR_R	GGACTACCAGGGTATCTAATCCTGTT	[17]
16S_qPCR_probe	(FAM) CGTATTACCGCGGCTGCTGGCAC (TAMRA)	[17]

**Table 2:** Homology of the amino acid sequence of SufC to *S. pneumoniae* D39.

Species	Accession Number	Identity (%)	Positives (%)	Gap (%)
*Streptococcus oralis*	CBZ00495	100	100.0	0.0
*Streptococcus mitis*	CBJ22162	99.6	99.6	0.0
*Streptococcus gordonii*	ABV10406	94.9	98.8	0.0
*Streptococcus sanguinis*	ABN45331	94.5	98.4	0.0
*Streptococcus salivarius* CCHSS3	CCB92513	90.2	97.3	0.0
*Streptococcus mutans* UA159 (serotype c)	AAN58017	90.2	96.1	0.0
*Streptococcus pyogenes* M1 GAS (serotype M1)	AAK33355	85.9	94.1	0.0
*Staphylococcus aureus* RF122	CAI80462	75.8	88.3	0.0
*Escherichia coli* K-12 MG1655	NP_416197	52.2	70.9	0.8

## Data Availability

Data supporting the results of this study are included in this paper. These data are not archived in any public repository.
